# Chemical Recycling of CFRP in an Environmentally Friendly Approach

**DOI:** 10.3390/polym16010143

**Published:** 2024-01-02

**Authors:** Asuka Sakai, Winarto Kurniawan, Masatoshi Kubouchi

**Affiliations:** 1 School of Materials and Chemical Technology, Tokyo Institute of Technology, 2-12-1, Ookayama, Meguro-ku, Tokyo 152-8552, Japan; kurniawan.w.ab@m.titech.ac.jp; 2 Nissan Motor Co., Ltd., 560-2 Okatsukoku, Atsugi 243-0192, Kanagawa, Japan

**Keywords:** CFRP recycling, epoxy resin decomposition, nitric acid, sodium hydrogen carbonate

## Abstract

A novel and environmentally friendly recycling approach for carbon-fiber-reinforced plastics (CFRP) was studied using not only nitric acid (HNO_3_) but also our chosen alkaline, sodium hydrogen carbonate (NaHCO_3_). The CFRP specimen was first immersed into 8 M HNO_3_ at 80 °C for 8 h, and then into 0.1 M NaHCO_3_ at 80 °C for 15 min to obtain resin-free recycled carbon fiber (rCFs). Using this new recycling method, it was shown that the recycling time was reduced to 8.3 h, whereas it originally took 24 h, as reported previously. It was shown that immersing the CFRP specimen into NaHCO_3_ caused a transesterification reaction with the remaining resin residue on the CF surface, which led to dissolving the resin into the NaHCO_3_ aqueous solution all at once. Additionally, NaHCO_3_ produced carbon dioxide gas while reacting with the resin residue; the CO_2_ gas physically helped removing the resin from the CF’s surface. Moreover, evaluating the physical properties of the rCFs demonstrated an improvement in fiber strength and adhesiveness to resin. Therefore, this recycling method was shown to be effective in recovering high-quality rCFs in a relatively short recycling period.

## 1. Introduction

Carbon-fiber-reinforced plastics (CFRPs) are receiving much attention, especially in high-tech industries such as aircrafts and automobiles. Due to its excellent material properties such as lightweight and high-strength [1], the global market for CFRPs, which was approximately USD 8.6 billion in 2020 [2], is expected to triple between 2020 and 2030 and grow continuously [2]. CFRP is a composite material, which is mold from carbon fibers and thermosetting resin. Due to its moldability and adhesiveness to resin, epoxy resins are mainly used for the thermosetting resin [3]. However, most of the CFRP waste is currently disposed of in landfills [4] as it is difficult to incinerate. Once it was tried to incinerate the CFRP waste, the unburned carbon fiber remained in the incineration residue, since carbon fiber has high “flame retardance”. An unburned carbon fiber included into fly ash shortened the electrodes of the electric dust collector, due to its high “electric conductivity”, and stopped the incineration facility [5]. Therefore, currently, about 1000 tons/year of CFRP waste generated mainly from the manufacturing process of the main wing of a Boeing 787 aircraft is landfilled [6]. Its disposal amount will increase more and more in the future, since many CFRP waste parts will be disposed from not only process scraps but also from aircrafts or automobiles which have reached the end of their life [7]. Therefore, establishing a technology for recycling CFRPs before they are disposed of in large volumes will contribute not only to realizing a carbon-neutral society but also to reducing ocean plastic pollution, since carbon fibers disposed of in landfills cannot biologically decompose [8].

Accordingly, much research on the recycling of CFRP focusing on recovering high-value carbon fiber has been conducted in recent years. Since there is a need to establish a recycling method with a low environmental burden, this study focuses on a recycling method using nitric acid. The nitric acid recycling method could decompose the epoxy resin contained in CFRPs under relatively moderate conditions (80 °C and atmospheric pressure) [9,10,11]. This recycling method enables the recovery of recycled carbon fibers (rCFs) and possibly the recovery of decomposed epoxy resin from nitric acid [9,10,11]. Additionally, an evaluation of the physical properties of rCFs revealed that the strength of the adhesion between the carbon fibers and resin, as well as the single carbon fiber, increased compared to virgin carbon fibers (vCFs), allowing for the recovery of high-quality rCFs [11]. Based on the reasons outlined above, this recycling method not only has a low environmental burden, but it also has the potential to realize a recycling-oriented society because it may enable the application of rCFs to CFRP products that require high performance and the recovered epoxy resin could be recycled as a virgin epoxy resin material. However, a previous lab-scale study confirmed that proceeding with the recycling from fully cured CFRP products required a time frame of 24 h, which might be a relatively long time on an industrial scale [11]. In order to industrialize this recycling technology, it is important to shorten the recycling time to make the business endeavor successful. Therefore, reducing the recycling time is a desirable goal. Methods that employ an organic solvent to swell the epoxy resin in the CFRP, followed by the immersion of the CFRP in an organic acid to progress the depolymerization reaction, have been reported to reduce the recycling time [12,13,14,15]. Alternative methods have also been reported where CFRPs are immersed in an organic acid aqueous solution along with treatment using ultrasonic waves or microwaves, which are intended to accelerate the resin depolymerization reaction of the acid [16,17,18,19]. The fastest recycling time was reported to be 3 h [18], though it could not compare in general since the manufactured condition and the type of epoxy resin used in CFRP are not the same. However, these methods not only require a large amount of energy during the recycling process due to using ultrasonic waves or microwaves, but also hinder recycling the resin because the resin will be decomposed into a very small molecule, which will be difficult to recycle as a virgin resin material.

**Figure 1:**
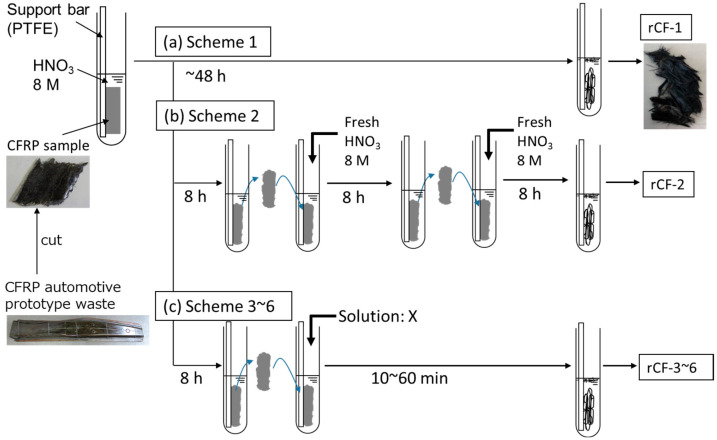
Decomposition and recovery process of carbon fiber from CFRP specimen. (**a**) Scheme-1: CFRP immersed in 8 M HNO_3_ for 5~48 h; (**b**) Scheme-2: CFRP immersed in 8 M HNO_3_ refreshed with HNO_3_ for every 8 h; (**c**) Scheme-3~6: CFRP immersed in solution X after immersion in 8 M HNO_3_ where solution X is NaOH, NaHCO_3_, sodium lauryl sulfate (SDS), poly ethylene glycol (PEG), and hexadecyltrimethylammonium bromide.

Therefore, we studied a recycling method that uses alkaline aqueous solutions to quickly dissolve the resin after using nitric acid under mild recycling conditions such as 80 °C. In our previous report, CFRP prepregs were immersed in nitric acid to progress the resin depolymerization, and then the CFRP specimen was immersed in the potassium hydroxide (KOH) aqueous solution. By immersing the specimen in a KOH aqueous solution, the side chains of the residual resin that remained on the carbon fiber’s surface were neutralized by the KOH and increased its solubility so that it easily dissolved in the aqueous solution [10]. However, KOH is a strong alkaline which requires sufficient attention when handling this chemical in recycling plants. Moreover, waste water containing KOH will have high alkalinity which cannot be treated in the waste water treatment plant and is necessary to be incinerated. Aiming to decrease the environmental burden of this method even further, this study investigated a new recycling scheme using sodium hydrogen carbonate, which is a weak alkaline that can be handled safely. In addition, the waste water from this recycling process has a possibility to be easily treated at the waste water treatment plant. This new approach of using sodium hydrogen carbonate as an alkaline aqueous solution was considered (Figure 1, Scheme-4) and compared to the original recycling scheme using only nitric acid (Figure 1, Scheme-1), and to other recycling schemes which used various chemicals (Figure 1, Scheme-2 to -6) under the same recycling conditions. Through this study, the optimal recycling scheme was discussed.

## 2. Materials and Methods

### 2.1. CFRP Materials

**Figure 2:**
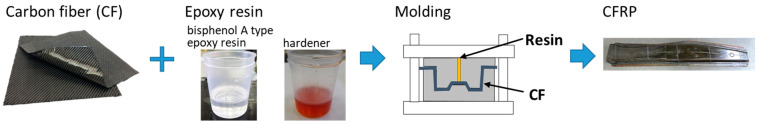
The composition of CFRP used in this research.

CFRP automotive prototype waste, which consisted of fabric-form vCFs and epoxy resin (bisphenol A type epoxy resin cured with polyamine), and mold by the Resin Transfer Molding (RTM) method were used as the CFRP samples (Figure 2). To proceed with the experiment in lab-scale, CFRP samples were crushed using a single-shaft crusher (EH-6090 (55 kW)), Horai Co., Ltd., Osaka, Japan) and the CFRP specimen was approximately 20 mm × 30 mm × 2 mm (length × width × thickness) in size (Figure 1).

### 2.2. Recycling Scheme of CFRP

(a) Scheme-1

**Figure 3:**
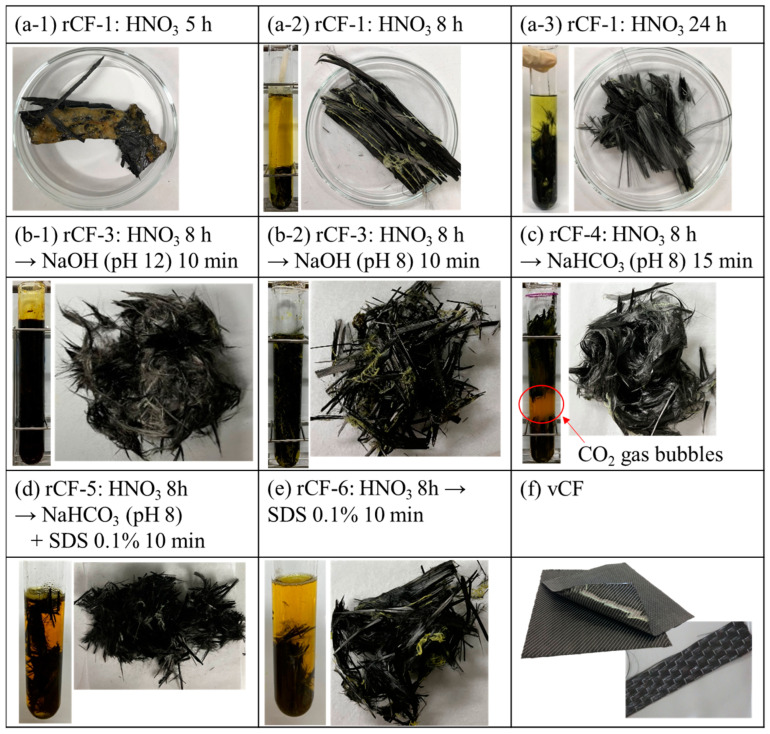
(**a**–**e**) Pictures of CFRP samples immersed in a test tube and the rCF collected under each solution condition described in each of the figures; (**f**) picture of vCF.

The CFRP specimen was immersed in a test tube (with inner and outer diameters of 6 and 8 mm, respectively) containing an aqueous solution of nitric acid (HNO_3_, Kanto Chemical Co., Inc., Tokyo, Japan, special grade) adjusted to 8 M by fixing the CFRP specimen into the test tube using a PTFE tube. The volume of HNO_3_ was adjusted to 10 mL for every 1 g of the CFRP specimen. The test tube was placed in a constant temperature bath at 80 °C and the epoxy resin was decomposed by the HNO_3_ over time, as shown in Figure 1. The solid residue, which was the carbon fibers, was collected from the test tube and rinsed using deionized water, and finally dried (at 80 °C for 48 h) to remove the moisture. Figure 3(a-1,a-2,a-3) show the photographs of the test tubes and the rCFs, which were collected 5, 8, and 24 h after immersion into the HNO_3_, respectively (hereafter rCF-1: HNO_3_ 5 h; rCF-1: HNO_3_ 8 h; and rCF-1: HNO_3_ 24 h).

(b) Scheme-2

In contrast to Scheme-1, we studied a reaction system in which the CFRP specimen was placed into a new HNO_3_ solution adjusted to 8 M every 8 h (Scheme-2). This scheme aimed to accelerate the resin decomposition process by replenishing the acid which was consumed through the resin decomposition reaction. As described in the procedure of Scheme-1, the CFRP specimen was immersed in HNO_3_ for 8 h, collected from the HNO_3_, and fully rinsed using deionized water. Subsequently, the specimen was immersed in a new HNO_3_ that was adjusted to 8 M for 8 h again, and collected and fully rinsed. Subsequently, the same operation was repeated and rCF was finally obtained after removing the residual moisture using a dryer (Figure 1b).

(c) Scheme-3

In Scheme-3 shown in Figure 1c, the CFRP specimen was first immersed in HNO_3_ (8 M) for 8 h, and when the resin decomposition rate reached approximately 80 mass%, the CFRP specimen was rinsed using deionized water. The volume of HNO_3_ was adjusted to 10 mL for every 1 g of the CFRP specimen, which was same as Scheme-1. Subsequently, the CFRP specimen was immersed in an alkaline aqueous solution under 80 °C. Sodium hydroxide (NaOH) (Kanto Chemical Co., INC., special grade) aqueous solutions adjusted to 1 × 10^−6^ M (pH 12), 1 × 10^−8^ M (pH 10), and 1 × 10^−10^ M (pH 8) were used as alkaline solutions to promptly dissolve the resin residue on the carbon fiber’s surface into the aqueous solution. The volume of NaOH was adjusted to 10 mL for every 1 g of the CFRP specimen, the same volume as HNO_3_. The specimen was collected after 1–60 min immersion in the NaOH and then rinsed and dried. Figure 3(b-1,b-2) show photographs of the test tubes and the rCFs, where the specimen was immersed in NaOH (pH 12) and NaOH (pH 8) for 10 min, respectively (hereafter rCF-3: HNO_3_ 8 h→NaOH (pH 12) 10 min and rCF-3: HNO_3_ 8 h→NaOH (pH 8) 10 min).

(d) Scheme-4

In Scheme-4 (Figure 1c), as compared with Scheme-3, a sodium hydrogen carbonate (NaHCO_3_) (Kanto Chemical Co., Inc., reagent special grade) aqueous solution, adjusted to either 0.01 M (pH 8.2) or 0.1 M (pH 8.3), was used for the alkaline aqueous solution. As described in the procedure of Scheme-3, the CFRP specimen was immersed in HNO_3_ (8 M) for 8 h, and then in NaHCO_3_ aqueous solution at 80 °C for 1–90 min. The volume of HNO_3_ and NaHCO3 was adjusted to 10 mL for every 1 g of the CFRP specimen, which was same as the scheme mentioned above. By immersing the specimen in an NaHCO_3_ aqueous solution for 15 min, the resin and nitric acid that remained on the carbon fiber’s surface reacted with NaHCO_3_, producing carbon dioxide gas bubbles (NaHCO_3_ + HNO_3_ → NaNO_3_ + H_2_O + CO_2_↑), and the resin-free rCF was collected (Figure 3c, hereafter rCF-4: HNO_3_ 8 h→NaHCO_3_ (pH 8) 15 min).

(e) Scheme-5

In Scheme-5 (Figure 1c), a surfactant was added to the NaHCO_3_ aqueous solution, with the other recycling conditions the same as in Scheme-4. In this recycling scheme, the effect of gas bubbles on the reaction of removing the resin residue from rCFs’ surface was considered, since it is known that surfactant helps to maintain gas bubbles in aqueous solutions [20]. Anionic, cationic, and nonionic surfactants were selected as the surfactant types, the measurements were performed under 0.1–1% concentrations, and the specimen immersion time was 1–60 min, as summarized in Table A1 (Appendix A). The rCFs were obtained using a procedure similar to the one described previously (Figure 3d).

(f) Scheme-6

In Scheme-6 (Figure 1c), as compared with Scheme-5, the specimen was immersed in HNO_3_ (8 M) for 8 h and then fully rinsed with deionized water. The CFRP specimen was then immersed in an aqueous solution only containing the surfactant at a concentration of 0.1–1% for 20–60 min (Figure 3e).

### 2.3. Method of Recovering Decomposed Resin

**Figure 4:**
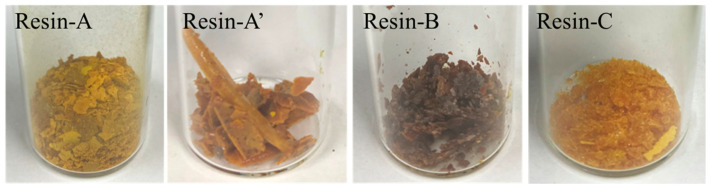
Pictures of the recovered decomposed resins. Resin-A from HNO_3_, in which the CFRP was immersed for 24 h in Scheme-1; Resin-A’ from HNO_3_, in which the CFRP was immersed for 8 h in Scheme-1; Resin-B from NaOH (pH 8) in Scheme-3; and Resin-C from NaHCO_3_ in Scheme-4.

The decomposed resin dissolving into HNO_3_ in Scheme-1 and alkaline in Scheme-3 and -4 was recovered through liquid–liquid extraction using ethyl acetate (Kanto Chemical Co., Inc., >99.3%) as an organic solvent. A total of 40 mL of ethyl acetate was mixed with the aqueous solution (20 mL), and the organic phase was then separated and collected using the separatory funnel. This operation was repeated two more times using 30 mL of ethyl acetate, respectively. The collected organic solvent containing the decomposed resin was neutralized with 20 mass% NaHCO_3_ until the pH reached 7. The organic solvent was removed using the evaporator, and then dried in the oven at 80 °C for 48 h. Finally, the decomposed resin shown in Figure 4 was collected.

### 2.4. Evaluation

#### 2.4.1. Time Change in Resin Decomposition Ratio

The resin decomposition rate *R*_d_ (mass%) of the CFRP specimen was calculated using Equation (1):

(1) $$R_{d}=\frac{\left(m_{0}-m_{t}\right)}{m_{0}\times F_{m}}\times 100$$

where *m*_0_ is the sample mass before immersion in HNO_3_, *m*_t_ is the sample mass of the collected rCFs, and *F*_m_ is the mass fraction of resin that is contained inside the CFRP specimen (40.0%) [5].

#### 2.4.2. Analysis of the Decomposed Resin

**Figure A1:**
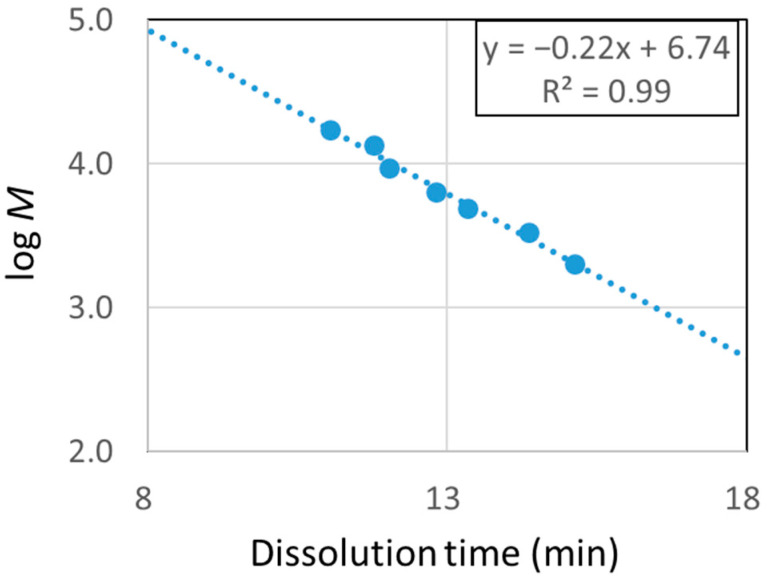
Calibration curve (molecular weight distribution of polystyrene measured with SEC).

Molecular weight distribution was measured using Size Exclusion Chromatography (SEC) (PU-980, JASCO Corporation, Tokyo, Japan). Tetrahydrofuran (THF) was used as the mobile phase. The average molecular weight of the decomposed resin was calculated using the calibration curve of polystyrene (Figure A1). Fourier Transform Infrared Spectroscopy (FT-IR) (FT-IR-8300, SHIMADZU CORPORATION, Kyoto, Japan) was used to analyze the functional groups of the decomposed resin, which were measured using the potassium bromide (KBr) disk method.

#### 2.4.3. Analysis of the Carbon Fiber Surface

The rCF surfaces were observed using scanning electron microscopy (SEM) (JSM-6510LA, JEOL Ltd., Tokyo, Japan).

#### 2.4.4. Adhesiveness of the Carbon Fiber and Resin

A microdroplet test was performed on vCF and rCFs to evaluate the adhesion between the carbon fibers and resin. The epoxy resin used in this test was the same as that used in CFRP products, and the microdroplet test was performed using the same curing conditions. Equipment for the evaluation of the interfacial properties of composite materials (model HM410, Tohei Sangyo Co., Ltd., Shizuoka, Japan) was used for the measurement. The maximum load *F* (×10^−3^ N) when pulling a single fiber out of a resin ball at a speed of 0.12 mm/min was measured, and the interfacial shear strength *τ* (MPa) was calculated using Equation (2):

(2) $$\tau =\frac{F}{\pi dL}\times 10^{3}\;$$

where *F* is the maximum measured load (×10^−3^ N), *d* is the carbon fiber diameter (specification value *d* = 7.0 μm), and *L* is the diameter of the resin ball (measured value of 50–80 μm).

The measurement was performed at least *n* = 20 times. The interfacial shear strength values when the resin ball size was *L*= 70 μm were calculated, since it has been reported that the interfacial shear strength *τ* is positively correlated to the diameter of the resin ball *L* [21].

#### 2.4.5. Tensile Strength of Carbon Fibers

The tensile tests for single vCFs and rCFs were performed in accordance with JIS R 7606, and the tensile strength and tensile modulus were evaluated. The single fiber was fixed to the paper [5] and fastened to a tensile tester (microstrength tensile tester, AcroEdge Corporation, Osaka, Japan), and the maximum load *F*’ (×10^−3^ N) at a pulling speed of 1.0 mm/min was measured. Additionally, the fracture strain *ε* (=Δ*λ*/*λ*) was calculated using the displacement prior to fracture Δ*λ* and the length of the carbon fibers *λ* (mm). Equations (3) and (4) were used to evaluate the tensile strength *σ* (GPa) and tensile modulus *E* (GPa):

(3) $$\sigma =\frac{4F^{\prime }}{\pi d^{2}}$$

(4) $$E=\frac{\sigma }{\varepsilon }$$

where *F*’ is the maximum measured load (×10^−3^ N) when a single fiber is fractured, *d* is the carbon fiber diameter (specification value *d* = 7.0 μm), and *ε* is the strain (=Δ*λ*/*λ*). The tensile strength measurements were performed at least *n* = 20 times.

## 3. Results and Discussion

### 3.1. Resin Decomposition Ratio of the Carbon Fiber and Surface Observation Results of Recycled Carbon Fiber

**Figure 5:**
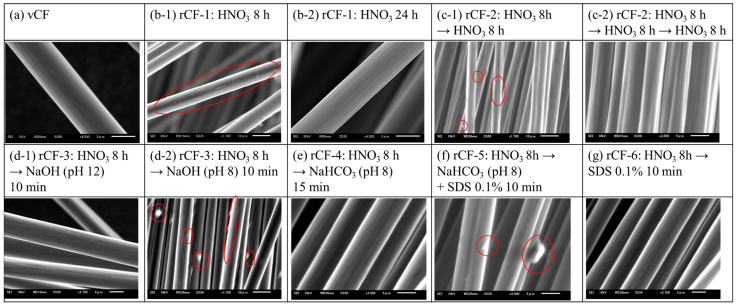
SEM images (SEI) of the surface of (**a**) vCF and (**b**–**g**) rCF-1~6, where the red circle represents the resin residues.

Table A1 lists the results of the calculated temporal changes in the resin decomposition rates of the recovered rCFs in each of the investigated recycling schemes (Scheme-1 to -6). A previous study reported that the resin decomposition rates in Scheme-1 were 76, 89, 95, and 95 mass%, for which the immersion times of the CFRP specimen in HNO_3_ were 8, 12, 24, and 48 h, respectively [11]. As shown in Figure 5(b-1), slight resin residue was observed; however, no resin residue was observed from the rCF shown in Figure 5(b-2).

In Scheme-2, no improvement in the resin decomposition rate was observed, as compared to Scheme-1 and as suggested in Table A1. This result indicates that a sufficient amount of HNO_3_ still remained to continue the reaction at 8 h even though the acid was consumed in the resin depolymerization reaction.

In Scheme-3 to -5, the resin decomposition ratio reached approximately 95% in a significantly short amount of time compared to Scheme-1. The resin decomposition rate in Scheme-3 reached 95% or more in just a 10 min immersion in NaOH (pH 12) (Figure 5(d-1)). The resin decomposition rate reached 95% or more in shorter processing time, such as 1 min when immersed in NaOH (pH 10). An excessively strong alkalinity was considered to hinder the permeation of NaOH through the decomposed resin sticking to the carbon fiber’s surface, which resulted in the slightly longer time requirement for the pH 12 NaOH solution to dissolve and remove most of the resin from the carbon fiber’s surface. Resin residue still remained on the carbon fiber’s surface of the rCFs after a 10 min immersion in pH 8 NaOH (Figure 5(d-2)). Finally, it took approximately 60 min to remove most of the resin residue and to reach *R*_d_ = 95%. The abovementioned findings indicate that pH 10 is the most appropriate condition for the reaction system in Scheme-3, which uses NaOH as the alkaline aqueous solution to remove the resin from the carbon fiber’s surface in a short amount of time.

In contrast, the resin decomposition rate in Scheme-4, in which a NaHCO_3_ aqueous solution (pH 8) was used, reached 95% in approximately 15 min (Figure 5e), which indicates that the required immersion time is a quarter of that when pH 8 NaOH is used. The test tube containing the CFRP specimen after immersion in NaHCO_3_ produced CO_2_ gas bubbles through the reaction with the HNO_3_ that remained on the carbon fiber’s surface (Figure 3c). Therefore, these CO_2_ gas bubbles presumably helped to physically separate the resin from the carbon fiber’s surface, allowing for the recovery of rCFs with no residual resin in a significantly short amount of time and in spite of the weak alkaline environment.

The resin decomposition rate in Scheme-5, either using an anionic, nonionic, or cationic surfactant, reached 95% or more in a short amount of time, which was approximately 10 min, even when the NaHCO_3_ concentration was very low (0.01 M). Although the surfactant concentration was very low (1.0%), the concentration was considered to be sufficient to form and sustain CO_2_ gas bubbles (Figure 5f).

Finally, the reaction system in Scheme-6 attempted to dissolve and remove the resin using only a surfactant; however, the resin decomposition rate did not improve significantly compared to Scheme-1 (Figure 5g).

The abovementioned findings demonstrate that the recycling scheme, which firstly decomposes the epoxy resin using nitric acid and secondly dissolves the resin using an alkaline, allows us to shorten the recycling time.

### 3.2. Consideration of the Mechanism to Remove Resin from the Carbon Fiber’s Surface by Using an Alkaline

The mechanism of the reaction between nitric acid or alkaline and epoxy resin was considered.

#### 3.2.1. Molecular Weight Distribution of Decomposed Resin

**Figure 6:**
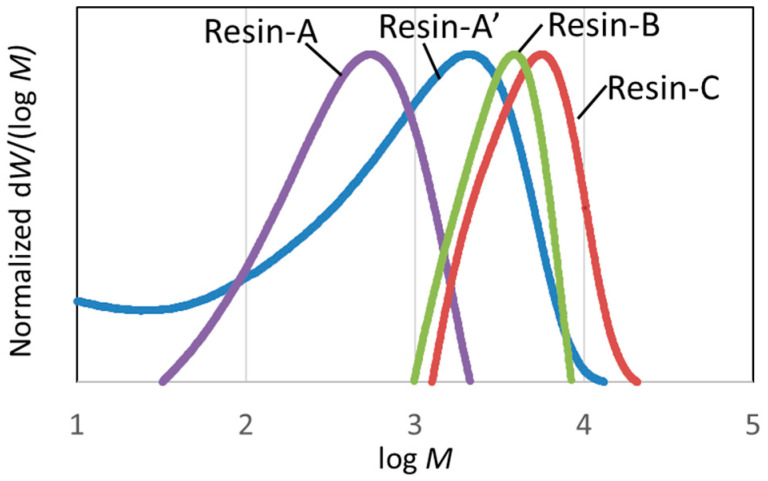
Molecular weight distribution of Resin-A, -A’, -B, and -C, measured using SEC.

**Figure 7:**
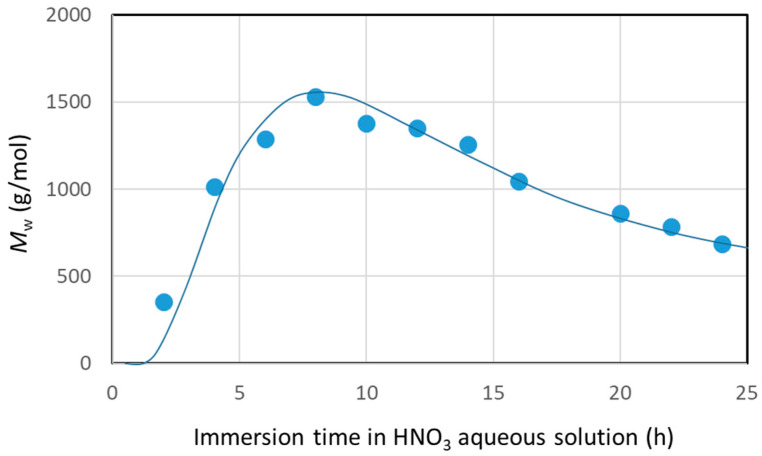
Average molecular weight of decomposed resin recovered from HNO_3_.

The molecular weight distributions of Resin-A, A’, B, and C, measured using SEC, are shown in Figure 6. Furthermore, the molecular weight distribution of decomposed resin, which was recovered from nitric acid in Scheme-1 when changing the immersion time of CFRP in nitric acid for 2 to 24 h, was measured. The average molecular weight *M*_w_, which was calculated using the calibration curve of polystyrene, is plotted versus the immersion time of CFRP in HNO_3_ (Figure 7). Figure 7 shows that, first of all, the epoxy resin decomposed and dissolved in HNO_3_ in a low-molecular-weight structure. The average molecular weight reached its peak when the immersion time reached 8 h, and the average molecular weight decreased when the immersion was continued. The average molecular weight showed *M*_w_ = 688 and *M*_w_ = 1531 for Resin-A and –A’, respectively. On the other hand, the average molecular weight for Resin-B and Resin-C was *M*_w_ = 4073 and *M*_w_ = 6044, respectively. Resin-B showed a value approximately four times higher than of Resin-A’, although in fact that Resin-B and C are the Resin A’ that has been subjected to further processing.

#### 3.2.2. FT-IR Measurement of Decomposed Resin

**Figure 8:**
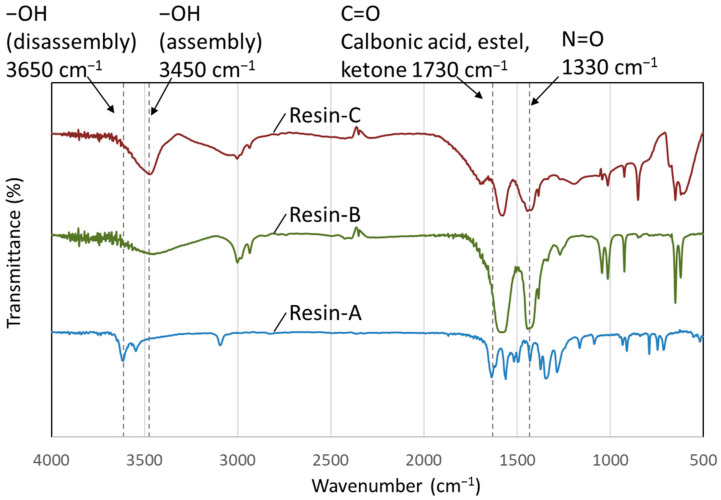
FT-IR spectra of decomposed resin measured using the KBr disk method.

The FT-IR spectra shown in Figure 8 confirmed that the peak originating from C=O was observed at around 1730 cm^−1^ in Resin-A; however, the peak was weakened in Resin-B and Resin-C. Secondly, a peak originating from -OH was observed in Resin-A at around 3650 cm^−1^. A similar peak was also observed in Resin-B and Resin-C; however, it was confirmed that the peak shifted to around 3450 cm^−1^. It was assumed that the decomposed resin with -OH functional groups gathering together through hydrogen bonds resulted in the peak shift.

#### 3.2.3. Resin Dissolution Mechanism Using an Alkaline

**Figure 9:**
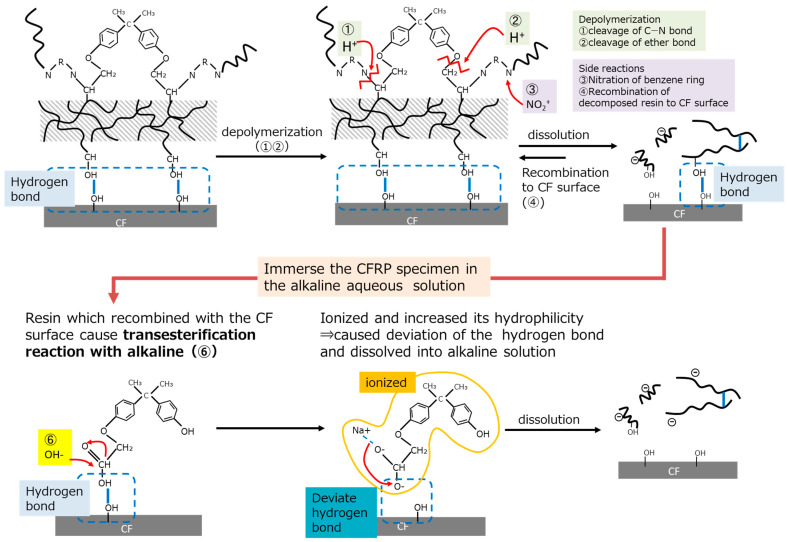
Schematic image of the decomposition and dissolution mechanisms of the epoxy resin with nitric acid and alkaline aqueous solutions.

According to the results of the decomposed resin analysis, the schematic images of the decomposition and dissolution mechanisms using nitric acid and alkaline are shown in Figure 9. First of all, it is reported that epoxy resin combines hydrogen bonds with polar functional groups which exist on the carbon fiber’s surface [22]. Thereafter, the nitric acid cleaved the C-N bond and ether bond of the resin [23], whilst the CFRP specimen was immersed in HNO_3_. Then, the resin which decomposed to a low molecular weight dissolved in HNO_3_, because the hydrophilicity increased due to the cleavage of the long-chain polymer. However, the decomposed resin that still had a high molecular weight recombined with the carbon fiber’s surface through hydrogen bonds, as it still had high hydrophobicity. Hence, when the CFRP specimen was only immersed in HNO_3_, it took a long time to obtain decomposed resin-free carbon fiber and for all the epoxy resin to be removed.

When the CFRP specimen was immersed in an alkaline aqueous solution, a weakening in the C=O peak was observed. Therefore, the alkaline caused a transesterification reaction with the epoxy resin so that the decomposed resin, even with a high molecular weight, ionized and increased its hydrophilicity, caused the deviation of the hydrogen bonds, and finally dissolved into alkaline solution. This assumption was supported by the results of the average molecular weight of the decomposed resin, which was recovered from the alkaline aqueous solution and showed a value four times higher compared to that recovered after a 24 h immersion in nitric acid solution. In addition, the decomposed resin recovered from the alkaline aqueous solution showed a peak shift in –OH functional groups in the FT-IR analysis, which suggests that several resin molecules dissolving in the alkaline aqueous solution might associate with each other.

Moreover, using NaHCO_3_ aqueous solution was effective in allowing for the recovery of rCFs with no residual resin in a significantly short amount of time, in spite of the weak alkaline environment. It produced CO_2_ gas bubbles through the reaction with the HNO_3_ that remained on the carbon fiber’s surface. This reaction presumably occurred at or close to the interface between carbon fiber and the resin, which was considered to help physically separate the resin from the carbon fiber’s surface. Therefore, even though implementing CO_2_ or micro bubbles into other recycling schemes, such as Scheme-3, which used NaOH as an alkaline, it was considered not to observe the same effect as recycling Scheme-3. This will be discussed in further research through additional experiments.

### 3.3. Mass Balance of the Input and Output Materials through Recycling Scheme- 4

**Figure 10:**
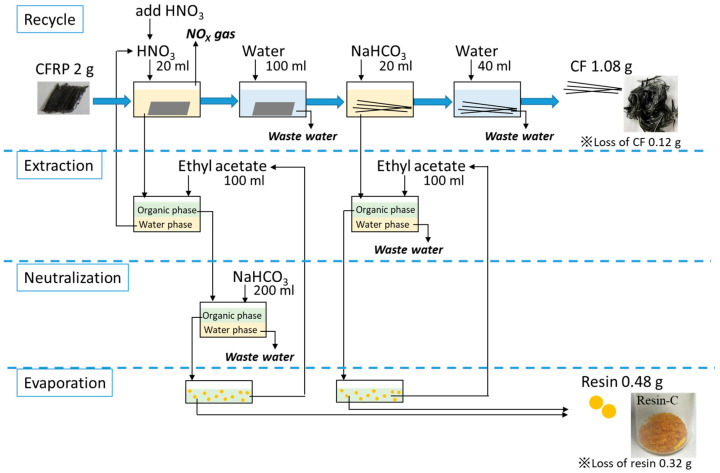
Schematic image of the mass balance of input and output through recycling process.

The mass balance of the input such as CFRP materials, chemicals, and water, along with the output such as recovered materials, discharged water, and exhaust gas are summarized in Figure 10. Considered simply, the mass balance was calculated based on 2 g of CFRP to be recycled.

First of all, as mentioned above, carbon fibers and decomposed resin could be recovered as a solid through this recycling process. The mass fraction of carbon fibers and resin that composed the CFRP specimen was 60% and 40%, respectively, as described in Section 2.4.1. The recovering ratio of rCF was approximately 90%, which had some loss that could not be collected through the operation of replacing the specimen in each recycling step; therefore, 1.08 g of rCF was collected from 2 g of CFRP. In the same way, the mass volume of recovered resin was calculated. The recovering ratio of resin showed 40%, which was quite low compared to the recovering ratio of rCF. The loss of resin was considered to occur through the extraction process that some decomposed resin remained into the water phase and could not transfer into the organic phase; however, this value could be improved by optimizing the extraction process. The collected resin was calculated to be 0.48 g. The collected resin could be reused as a material virgin epoxy resin material, which was shown in our previous research [9,10,11].

In terms of liquids, the water used for washing the specimen after the HNO_3_ and NaHCO_3_ immersion process could be treated in a waste water treatment plant. Additionally, NO_X_ gas will exhaust through the chemical reaction between HNO_3_ and resin. The HNO_3_ used for resin depolymerization could be recycled because the decomposed resin will be extracted from HNO_3_. However, it is necessary to add new HNO_3_ and to adjust the HNO_3_ concentration to 8 M, since acid is consumed through the recycling. The discharged NaHCO_3_ used for the recycling process and neutralization needs to be treated as waste water.

Therefore, only waste water, which could be treated in the waste water treatment plant, will be discharged through this recycling scheme. The energy consumption in the waste water treatment process is 3.64 CO_2_/m^3^-water [24,25]. Since its value varies depending on the treatment method, it is obviously a small value. Moreover, other chemical recycling methods using organic solvent could not recover decomposed resin from the solvents. Therefore, the solvents used for recycling are difficult to be reused since the decomposed resins remain in the solvents. To discuss the actual environmental impact of this proposed new recycling scheme, further research on the exact energy consumption in detail is necessary. However, this recycling method could be expected as an environmentally friendly method due to less output that could not be reused.

### 3.4. Physical Properties of Carbon Fiber

In our previous report, high-quality rCFs were recovered from recycling Scheme-1 [11]. This section compares the physical properties of rCF-4 (rCFs recovered using recycling Scheme-4) and vCF to determine the efficacy of Scheme-4 as a new recycling method, which showed a significant reduction in its recycling time.

#### 3.4.1. Interfacial Shear Strength between Carbon Fiber and Resin

**Figure 11:**
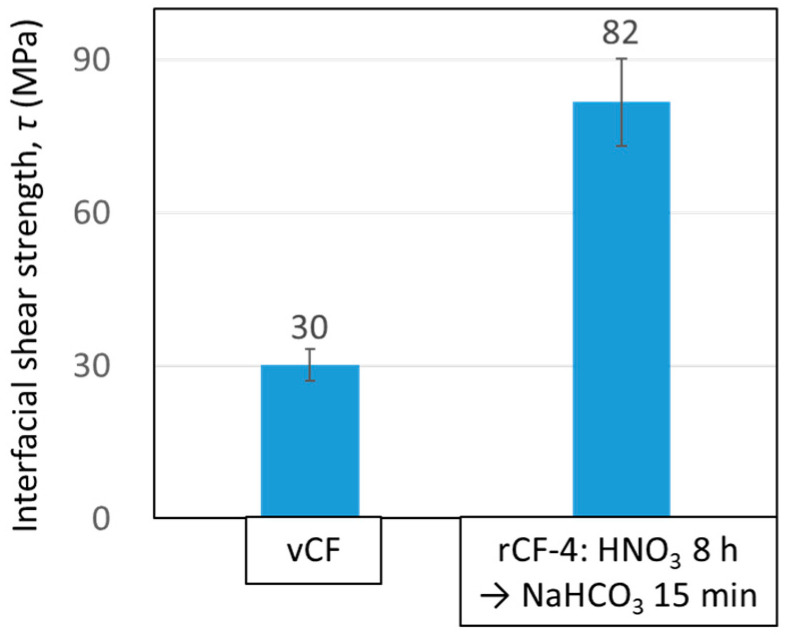
Interfacial shear strength between carbon fiber and resin measured with microdroplet tests for a data plot with a resin droplet size of *L* = 70 μm for vCF and rCF-4.

Microdroplet tests were performed on vCF and rCF-4, and the results are shown in Figure 11. The interfacial shear strength of rCF-4 was *τ* = 82 ± 8.6 MPa, which is 2.7 times higher than that of vCF (*τ* = 30 ± 3.1 MPa). A previous study confirmed that nitric acid formed polar functional groups on the carbon fiber’s surface during the recycling process, which improved its adhesiveness to resin [11]. An elemental analysis of the carbon fiber’s surface was conducted using XPS to determine whether the formation of polar functional groups on the carbon fiber’s surface similarly increased in the newly studied Scheme-4. Table A2 lists the results of the functional group composition ratios, which were obtained via XPS measurement. The functional groups that are related to resin bonding are shown in red font. rCF-4 contained 8.3% functional groups that were derived from nitrogen, specifically from the nitro and amino groups. The functional group content in vCF was approximately 1%, which suggests that the nitric acid caused these nitro and amino groups to form on the carbon fiber’s surface. Moreover, the vCFs contained 19.5% oxygen, whereas rCF-4 contained 21.5% oxygen. This result suggests that, although the recycling process removed the sizing agent, new functional groups formed on the surface. The abovementioned results show that the functional groups related to resin bonding were also formed on the carbon fiber’s surface during the recycling process in the newly studied Scheme-4, which led to the increase in the interfacial shear strength with the resin.

#### 3.4.2. Tensile Strength of Carbon Fiber

**Figure 12:**
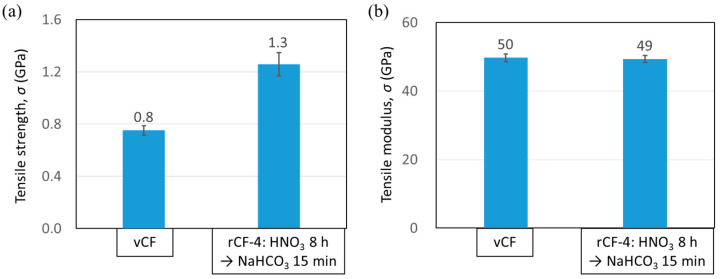
(**a**) Tensile strength and (**b**) tensile modulus of vCF and rCF-4 measured using a single-fiber fragmentation test.

**Figure A2:**
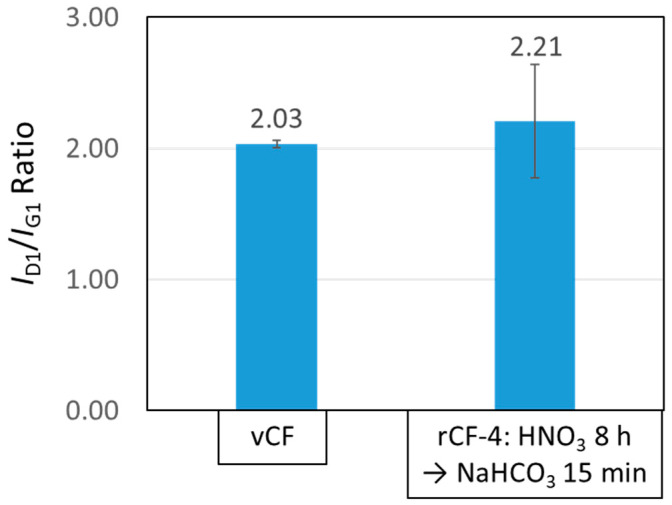
Intensity ratio of D1 band and G1 band obtained from Raman spectroscopy analysis measured on the surface of vCF and rCF-4.

Single-fiber tensile tests were performed on vCF and rCF-4, and the tensile strength *σ* and tensile modulus *E* are shown in Figure 12a,b, respectively. The tensile strength of rCF-4 was *σ* = 1.3 ± 0.09 GPa, which is 1.6 times higher than that of vCF. The results of the tensile modulus did not significantly differ between vCF and rCF-4, suggesting that there was no substantial structural change in the graphite structure constituting the carbon fibers [11] (Figure 12b). Raman spectroscopy was performed for vCF and rCF-4 in order to confirm the graphite structure’s crystallinity. The intensity ratio of the D1 peak area to the G1 peak area (*I*_D1_/*I*_G1_) was recorded, and the results are shown in Figure A2. A relatively large variation was observed in the rCF-4 data (standard deviation of the data: ± 0.44). However, there was no significant difference between vCF and rCF-4 in the *I*_D1_/*I*_G1_ ratio itself, implying that the graphite structure did not become disordered during the recycling process.

## 4. Conclusions

This study investigated a new recycling system using sodium hydrogen carbonate, which is a weak alkaline that can be handled safely. This new recycling scheme was investigated in order to reduce the recycling time in an environmentally friendly recycling method. The main findings were as follows:

(1) A CFRP specimen immersed in nitric acid for 8 h followed by immersion in sodium hydrogen carbonate aqueous solution for 15 min resulted in a reduction in the recycling time, whereas it originally took 24 h.
(2) This new recycling scheme was effective at quickly removing the epoxy resin from the CF surface, since the alkaline reacted with resin and dissolved it in an aqueous solution even if the epoxy resin remained in a long chain. Moreover, sodium hydrogen carbonate was the most effective alkaline to use because it produced carbon dioxide gas through the chemical reaction between sodium hydrogen carbonate and nitric acid, which helped removing the resin physically by the carbon dioxide gas bubbles. This was the first research that elucidated this mechanism.
(3) The physical properties of rCFs, such as the fiber strength as well as the interfacial shear strength between the CF and the resin, which were recovered through this new recycling scheme expressed higher value than those of vCFs.5. Finally, the abovementioned findings show that the recycling method using sodium hydrogen carbonate, which can be safely handled, is an effective recycling method that reduces recycling time and enables the recovery of high-quality rCFs.

## Data Availability

Data are contained within the article.

## Appendix A

**Table A1:** Recycling condition and resin decomposition ratio under each recycling Scheme-1~6.

Recycling Scheme	1st Step			2nd Step						Recycled Carbon Fiber	
	Type of Solution	Conc.	Immersion Time	Type of Solution	Conc.	Immersion Time	Additives	Conc.	Immersion Time	Type of rCF	*R*_d_ (mass%)
Scheme-1	HNO_3_	8 M	5 h							rCF-1	61.0
			8 h								75.8
			9 h								80.1
			12 h								89.1
			24 h								94.9
			48 h								95.3
Scheme-2	HNO_3_	8 M	8 h	HNO_3_	8 M	2 h				rCF-2	77.9
						4 h					82.8
						6 h					88.1
						8 h					94.8
						16 h					95.2
Scheme-3	HNO_3_	8 M	8 h	NaOH	pH 12	1 min				rCF-3	91.3
						10 min					98.3
					pH 10	1 min					96.4
					pH 8	1 min					81.3
						10 min					82.0
						20 min					92.4
						60 min					96.6
Scheme-4	HNO_3_	8 M	8 h	NaHCO_3_	0.1 M	1 min				rCF-4	81.3
						5 min					89.0
						10 min					92.5
						15 min					96.4
						20 min					98.5
						30 min					99.6
					0.01 M	90 min					96.6
Scheme-5	HNO_3_	8 M	8 h	NaHCO_3_	0.01 M	-	SDS	0.10%	10 min	rCF-5	85.0
									60 min		99.0
								1.0%	10 min		97.0
							PEG	0.10%	60 min		95.9
								1.0%	10 min		97.6
							PEG	0.10%	60 min		96.9
								1.0%	10 min		99.7
							SDS, PEG	0.1% each	1 min		92.6
								0.1% each	5 min		91.5
								0.1% each	10 min		96.9
							Cationic surfactant	0.10%	60 min		85.4
								1.0%	10 min		97.9
Scheme-6	HNO_3_	8 M	8 h	-			SDS	0.10%	10 min	rCF-6	81.5
									60 min		81.6
								0.30%	20 min		80.1
							PEG	0.10%	60 min		81.8
							PEG	0.10%	60 min		82.5

**Table A2:** Presence ratios of functional groups on the surface of vCF and rCF-4, measured via XPS, whose polar functional groups combined hydrogen bonds with epoxy resin, are shown in red font.

		vCF	rCF-4
C (%)	CH_n_	45.1	35.8
	-C-OH, -C-O-C	27.8	28.5
	>C=O	2.8	3.3
	-CO-O-	0.8	1.6
	-C=C-	0.7	0.8
	Total	77.2	70.1
N (%)	-NH_2_	0.0	3.3
	-NO/NH_4_	1.2	0.9
	-NO_2_	0.0	4.1
	Total	1.2	8.3
O (%)		19.5	21.5
Others (%)		1.9	0.2
